# Flexible resource use strategies of a central-place forager experiencing dynamic risk and opportunity

**DOI:** 10.1186/s40462-019-0168-2

**Published:** 2019-08-02

**Authors:** Kira L. Hefty, Kelley M. Stewart

**Affiliations:** 1 Department of Biology, University of Nevada, 1664 N. Virginia St. Reno, Reno, NV 89557 USA; 2 Department of Natural Resources and the Environment, University of Arizona, 1064 East Lowell Street, Tucson, AZ 85719 USA; 3 Department of Natural Resources and Environmental Science, University of Nevada, Reno, 1664 N. Virginia St. Mail Stop 186, Reno, NV 89557 USA

**Keywords:** Resource selection, Trade-offs, Movement behavior, Small mammal, GPS, High-resolution

## Abstract

**Background:**

Movement decisions made in space and time define how wildlife meet competing extrinsic and intrinsic demands to maximize fitness. Differential selection of resource patches provides one example of how to measure how animals balance conflicting demands. We hypothesized that individual spatial selection of patch types between dynamic seasons would signify flexible strategies used to minimize risk and optimize foraging efforts.

**Methods:**

We used data collected from GPS loggers on golden-mantled ground squirrels (*Callospermophilus lateralis*) to model selection or avoidance of resources in two seasons of seed availability and one season in which no seeds were available. Movement decisions were measured in short-term discrete time intervals using high resolution location data. Selection or avoidance of specific resource features that entail fitness consequences were then assessed using resource selection functions.

**Results:**

Seasonality of food availability, food type, and spatial distribution of food largely influenced how individuals selected resources within their home ranges. Overall, when seeds were available, individuals mediated risks of predation and loss of food by using patches closer to refuge and selected intermediate distances to the burrow. When food was not available, individuals minimized exposure to heightened risk by staying close to the burrow and avoiding riskier patch types.

**Conclusions:**

Results indicate that individuals used flexible, dynamic strategies to select habitat patches which may allow them to balance conflicting seasonal demands. Advances in GPS technology for research of small mammals provide greater insight into how prey species in high risk environments differentially use resources to minimize risk and maintain fitness.

## Background

All animals experience dynamic physiological and environmental demands and must adjust their activity patterns to minimize associated costs and increase fitness. Fulfilling energetic requirements, limiting competitive interactions, avoiding predation, searching for mates, and minimizing exposure to unfavorable abiotic elements are some of the competing demands that influence how species use resources within their environment [1]. The level of risk (i.e. cost to fitness) individuals perceive in response to these demands is not temporally or spatially constant, and often risk is compounded by simultaneously occurring demands [1, 2]. To appropriately respond to these dynamic conditions, individuals must differentially use movement paths and select resources that will minimize perceived risk.

Appropriate methods to analyze how individuals perceive and respond to risk associated with conflicting demands remain debated in literature [3, 4]. Many studies have used experimental methods to understand bilateral trade-offs in behavioral responses [5, 6]. For example, giving-up densities (GUDs) measure trade-offs between behaviors such as foraging effort and predator avoidance by measuring density of food left in a patch or experimental food trays as an indicator of the cost of foraging under predation risk [1, 7, 8]. GUDs assume perceived predation risk is greater with increasing amounts of food left by an individual, indicating the point at which the cost of foraging exceeds the energetic benefits. This approach has been criticized for its assumption that individuals assess food consistently among patches in time and space and disregards additional extrinsic and intrinsic factors that also may influence an individual’s decision to leave a patch [4, 5, 9, 10].

Animal movement is inherently driven by extrinsic and intrinsic factors. Patterns in movement reveal how individuals select resources to fulfill energetic requirements, minimize exposure to costly abiotic elements, and reduce predation and mortality risk [11, 12]. It is well documented that wildlife species will alter corridor use, daily activity patterns, and migration routes to minimize risk [12–14]. Recent advances in GPS technology and analysis methods have allowed for detailed inference regarding animal movement. These methods can provide valuable information regarding decisions individuals make in heterogeneous environments and how those decisions may change dependent on dynamic conditions within their environments. For example, differential selection of resources driven by food quality and quantity within and between seasons may be evident as shifts in movement paths between seasons as individuals select different habitat patches [15, 16]. Likewise, avoidance of a perceived risk, such as predator threat, may be evident as a reduction in density of location data in space or time where perceived risk is highest [17]. Extrinsic risks can be mediated by selecting safer corridors (i.e. corridors with ready access to refuge) or by modifications in individual behavior, such as periodic episodes of antipredator vigilance [3, 17, 18]. Given the intrinsic variability and uncertainty in heterogeneous environments, this flexibility in resource selection and behavior is critical for individual survival.

Golden-mantled ground squirrels (*Callospermophilus lateralis*) are an excellent study species to analyze how individuals use differential selection of resources to balance dynamic, conflicting demands. *C. lateralis* are ubiquitous in granivore-driven ecosystems in mountainous regions of western North America. Like other central-place foragers, *C. lateralis* must minimize the energetic cost of foraging while maximizing energy gained from food gathered [19, 20]. Additionally, *C. lateralis* are solitary, and individuals must minimize costs associated with dynamically changing demands such as predator pressure, exposure to abiotic elements in unsuitable matrices, and competitive interactions [21–23]. Adjusting activity patterns and movement paths is crucial to balance these risks, particularly in a system where food availability is seasonal and unpredictable year to year due to annual drastic variation in seed crop abundance [24, 25]. *C. lateralis* use a larder-hoarding strategy, in which they place all their seeds in a burrow and defend that burrow from sympatric granivore species [26, 27]. Competing yellow-pine chipmunks (*Tamias amoenus*) scatter-hoard seeds and use their acute olfactory sense to pilfer from caches made by conspecifics and other species [28]. *C. lateralis* cannot reciprocate this pilfering behavior, which makes larder-defense their primary strategy to store food [27, 28]. Despite the importance of defending the larder, individuals must often forego larder defense to participate in other essential activities, including foraging. When individuals leave the burrow, they not only leave the larder vulnerable to competitors, but also incur greater levels of direct physical risk, such as predation. Therefore, *C. lateralis* must effectively evaluate and appropriately respond to competing risks to maximize fitness.

Herein, we address how *C. lateralis* use flexible strategies to balance conflicting demands of predator avoidance, larder-defense, and foraging. Resource selection functions (RSFs) were used to identify differential selection of resources between seasons of seed availability and one season when seeds were not available. Though, RSFs have most commonly been used in studies of large mammals, they can be very helpful in determining resource use by small mammals that experience unique habitat constraints [29–32]. Assuming an overwhelming necessity of needing to forage in an environment of exceptionally unpredictable and limited food availability, we predicted that: 1) In all seasons, *C. lateralis* should select foraging sites closer to the burrow to protect the larder from pilferers and to reduce the risk of predation, 2) During seasons of seed availability, *C. lateralis* should select patches in which probable encounter rate with food is highest, and 3) When individuals are away from the burrow, they should remain within close proximity to points of refuge, such as boulders and stumps, to escape predators.

## Methods

### Study site

Our study area was located in the semiarid eastern Sierra Nevada, where seed availability is ephemeral and particularly limited [33, 34]. We conducted the study within a 0.43 km^2^ area in the Whittell Forest and Wildlife Area in Little Valley, Washoe County, 30 km south of Reno, Nevada, USA (39°15′0″N, 119°52′35″W). This study site is owned by the University of Nevada, Reno, and comprises 1073 ha at an average elevation of 1975 m. Dominant woody vegetation includes Jeffrey pine (*Pinus jeffreyi*), lodgepole pine (*Pinus contorta*), antelope bitterbrush (*Purshia tridentata*), greenleaf manzanita (*Arctostaphylos patula*), tobacco bush (*Ceanothus velutinus*), and Sierra bush chinquapin (*Castanopsis sempervirens*). The portion of Little Valley selected for this study had a dominant open tree canopy of Jeffrey pine and a patchy understory of antelope bitterbrush. Those two species of plants were the only seed-producing plants in the site to support populations of *C. lateralis* and granivorous competitors. Seeds are the dominant source of food for granivores in this study site and therefore dependence on this food source is much higher than other scarce opportunistic encounters with other food sources. Bitterbrush seeds, when available in July, were restricted to stands of shrubs existing primarily in clumped distribution beneath open (e.g., no overstory cover) canopy. Jeffrey pine seeds were randomly scattered by the wind in early September. Availability of both bitterbrush and Jeffrey pine seeds was abundant during the time of the study. Primary predators of *C. lateralis* during the summer and fall in this region primarily consisted of birds-of-prey.

### Animal capture and handling

During July–September, 2014, 9 adult *C. lateralis* (five males and four females) were selected for study (Table 1). Most individuals were used more than once, dependent on researcher ability to consistently recapture those individuals. Squirrels were captured in Tomahawk model #102 live traps (Tomahawk Live Trap Co., Hazelhurst, Wisconsin) and handled in accordance with a protocol approved by the Institutional Animal Care and Use Committee (UNR IACUC, #A07/08–30) that was in keeping with guidelines established by the American Society of Mammalogists for use of wild mammals in research [35]. GiPSy 5 global positioning system (GPS) loggers (5 g, 17 × 12 × 4 mm) supplied by TechnoSmart Europe Srl (Colleverde, Italy), and very high frequency (VHF) transmitters (0.8 g) supplied by Advanced Telemetry Systems Inc. (Isanti, Minnesota) were used to track individuals and retrieve them. Telemetry transmitters were epoxied to the side of each GPS logger, and loggers were attached to squirrels using zip-ties threaded through hollow fabric cords. Individuals on average weighed 165 ± 24 g.

**Table 1:** Capture, home range size, and GPS fix summary for individuals used in a 2014 study of resource selection of golden-mantled ground squirrels (*Callospermophilus lateralis*) in the Whittell Forest and Wildlife Area, NV, USA. Season represents differing availability of food items for individuals

Season	Individual ID	GPS Fix Total	Available: Used Ratio	95% Kernel Home Range Size (m^2^)
Bitterbrush	F1	1188	3:1	103,268
Bitterbrush	M1	866	3:1	30,880
Bitterbrush	M2	605	3:1	18,087
Bitterbrush	M3	250	3:1	11,010
Bitterbrush	M4	196	3:1	52,363
Bitterbrush	M5	157	3:1	82,090
Interval	F2	698	3:1	29,704
Interval	F3	1035	3:1	20,482
Interval	M2	73	3:1	18,087
Interval	F4	1768	3:1	23,041
Interval	M3	92	3:1	11,010
Interval	M4	122	3:1	52,363
Interval	M5	171	3:1	82,090
Pine	F2	196	3:1	29,704
Pine	F1	173	3:1	103,268
Pine	F3	69	3:1	20,482
Pine	M2	23	3:1	18,087
Pine	M4	195	3:1	52,363
Pine	M5	161	5:1	82,090

Data were collected in three different seasons. Those seasons were delineated by timing and type of seeds that were available: 1) bitterbrush season (bitterbrush seeds available on shrubs July 21–31, 2014), 2) interval season (no plants producing seeds, August 1–September 11, 2014), and 3) pine season (Jeffrey pine seeds available, September 12–19, 2014). GPS loggers were programmed to collect fixes on one of two schedules: 5 rapid fixes (i.e. 1 fix per second for 5 s) at 5 min intervals or 1 fix every 10 s. The 10 s fix collection was initially used to gain more accurate locations per fix, but was subsequently changed to every five minutes with rapid fixes to save battery and maintain accuracy by averaging the locations of the five rapid fixes. On these schedules, the battery lasted for 3–4 days. After that time, loggers were retrieved from the squirrels and data were downloaded.

### Habitat modeling

All GPS locations collected from all individuals were used to create 95% fixed kernel density home range polygons using the Geospatial Modelling Environment [36]. Home range estimates were smoothed using the least squares cross-validation (LSCV), which is associated with the least biased estimates of home range size, particularly with smaller sample sizes [37]. All home range polygons were imported into ArcMap 10.3 and inspected for accuracy and to ensure outlying points did not cause unrealistic elongation of the estimated home range.

A vegetation raster with 1-m resolution was created in ArcMap 10.3 that described three dominant canopy cover types: open, pine, and antelope bitterbrush. Open canopy was categorized by no overstory or understory vegetative cover. Pine canopy was categorized by no bitterbrush understory and pine as an overstory cover. Bitterbrush canopy was categorized by a dominant understory cover of bitterbrush, regardless of whether there was an overstory cover of pine or not. Open and pine canopy cover were defined via digitization using 1-m resolution imagery from the National Agriculture Imagery Program. Our study site was classified as 5% primary bitterbrush canopy cover, 50% Jeffrey pine cover, and 45% open canopy. In the field, bitterbrush cover, burrows, and refuge locations were mapped at the study site using a Trimble GeoExplorer 7x. Bitterbrush shrubs were mapped as a continuous stand if < 1 m apart. Refuge locations consisted of stumps, boulders, and fallen logs under which rodent tunnels were located. These locations were mapped as either points (< 1 m in diameter) or polygons (> 1 m in diameter). If refuge locations such as boulders were < 1 m apart, they were mapped as a continuous polygon. Distance to the nearest refuge area was calculated using the Near tool in ArcMap 10.2 [38]. Separate rasters were created for canopy cover and distance to the home burrow. Distance from the home burrow was represented using a Euclidean distance raster with each burrow as the point of origination.

Resource selection functions (RSFs) were used to determine the probability of selection or avoidance of resources [39]. RSFs are often used to inform movement connectivity analyses and are flexible in their ability to analyze changes in habitat selection over time. Rather than using a movement-based model such as a step selection function, we chose to implement the RSF to model resource use because we deemed it a more appropriate method to accurately measure macroscopic space use while considering the small spatial scale used by study individuals, observed above-ground behavior of individuals, and the nature of the unequal sample intervals between fix locations [40]. To account for the assumption made by RSFs that the study area is entirely available to each individual at any time step, we selected individuals whose home ranges did not include seasonal or long-term barriers to movement as well as areas in which resources could feasibly be exploited year-round by each individual at any time [41]. Field observations and live trap grids were used to determine resource accessibility for each individual prior to the study. To create the resource selection function, categories of used and available points were designated [42, 43]. Locations of squirrels obtained from GPS data loggers represented habitat used by each squirrel (used points), while habitat available to that squirrel was represented by creating a set of random points to represent availability. Random points were generated within each home range for each squirrel generally at a 1:3 ratio of used to randomly generated points. Random points were generated so that they proportionally represented the vegetative cover types within the home range. When used points were few (< 60) or random points did not proportionally represent the underlying vegetation within the individual’s home range, we increased the number of random points to 1:5 ratio to appropriately characterize available resources. Raster values for distance to burrow, distance to refuge, and vegetative canopy cover type (i.e. open, bitterbrush shrub cover, or pine tree cover) were extracted for both used and random points. Non-parametric Kruskal-Wallis tests were used to analyze differences among distance to burrow and distance to refuge between seasons. A post-hoc Dunn’s test was used to test pairwise comparisons between seasons.

Resource selection was evaluated for all individuals at the scale of the home range for each individual (third order scale) [39]. To represent variation in selection of resources among seasons, we created three different mixed-effect logistic regression models, one per season [44–46]. For each season, we modeled canopy cover type as a categorical covariate with three levels (pine, open, and bitterbrush) and distance to refuge and distance to the burrow as continuous covariates. All statistics were calculated using the lme4 package in R version 3.1.2 [47–49]. Individual squirrels were modeled as a random effect to account for variation in number of locations collected among individuals [47, 48]. Non-linearity was graphically inspected for continuous variables using frequency histograms. When non-linearity was suspected, continuous variables were represented as both linear and non-linear (quadratic) terms [50]. Model selection was performed using Akaike Information criterion adjusted for small sample sizes (AICc) [51]. We examined models closely for the presence of uninformative parameters and selected the model which best fit our data [52, 53].

Additionally, continuous variables were standardized to allow for direct comparison among parameter estimates [54, 55]. For the discrete variable of canopy type, pine canopy was chosen as the intercept because it comprised the majority of the study area. Additionally, most individuals make their burrows under open canopy and would not use pine canopy as shelter while trying to avoid a predator [56]. For discrete variables (i.e. vegetative canopy cover) resulting positive model coefficients indicated selection of a covariate while negative values indicated avoidance of a covariate [55]. For continuous variables representing distance to a resource (i.e. refuge or the burrow), negative model coefficients indicate selection for shorter travel distances because probability of selection decreases as distance to a point of interest increases. Alternatively, positive coefficients indicate avoidance of a resource of interest because probability of selection increases as distance from a resource increases.

## Results

All seasons were significantly different from one another for both distance to burrow (Kruskal-Wallis chi-squared = 180.36, df = 2, *p*-value< 0.001) and distance to refuge (Kruskal-Wallis chi-squared = 242.06, df = 2, *p*-value< 0.001). In general, individuals traveled farthest from the burrow during the bitterbrush season (66 ± 52 m) and remained relatively close to the burrow during the interval season (48 ± 26 m) and Jeffrey pine season (46 ± 57 m). Individuals were active closer to refuge locations on average during the bitterbrush season (9 ± 9 m) and Jeffrey pine season (7 ± 16 m) and were active farther from refuge locations during the interval season (12 ± 13 m). Therefore, separate models were run for each season.

### Resource selection

**Fig. 1:**
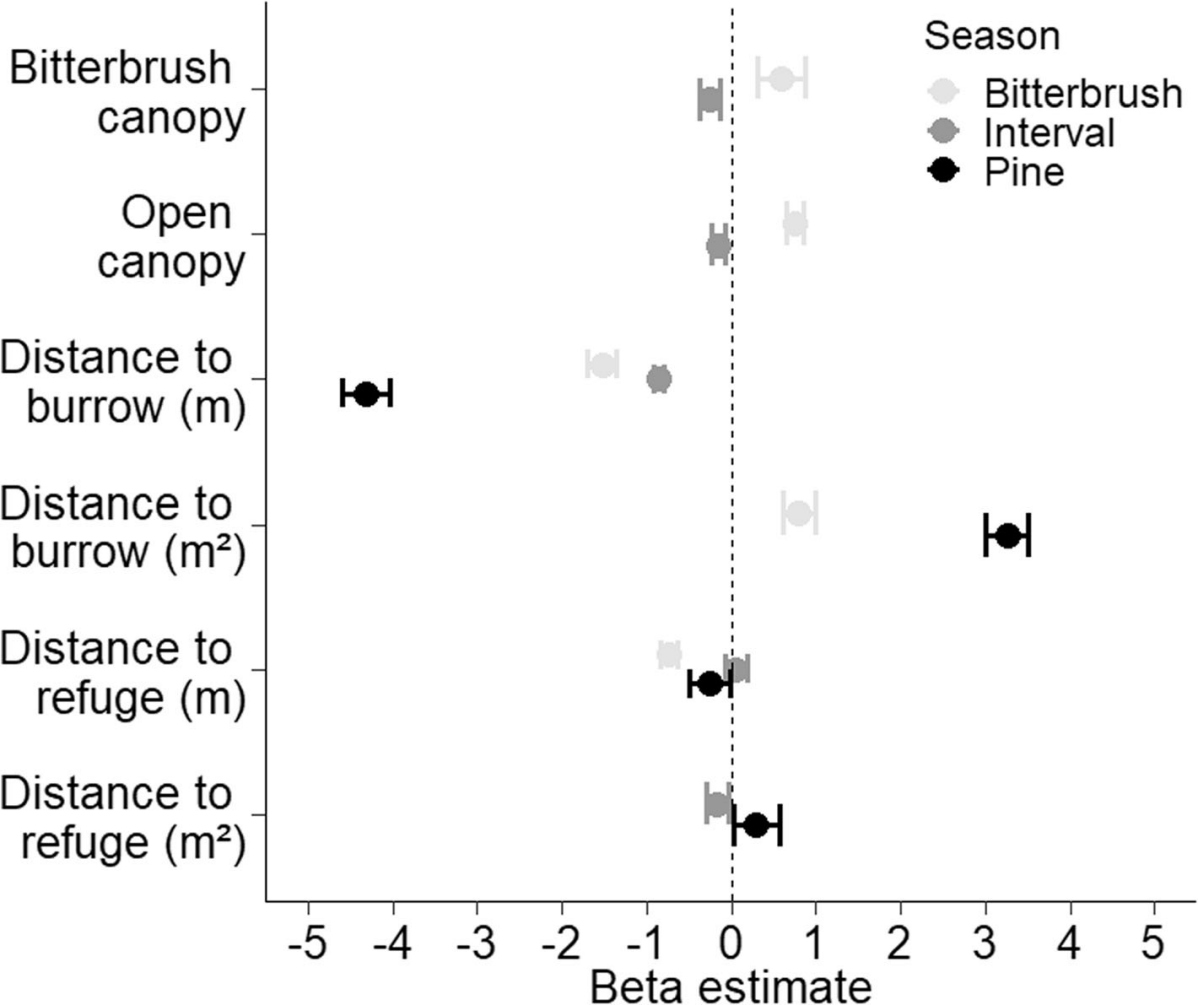
Model generated beta estimates with 85% confidence intervals from an analysis of resource selection by *Callospermophilus lateralis* relative to three seasons of food availability: bitterbrush, interval (no seeds available), and pine. Selection for parameters along the y-axis are represented by positive values along the x-axis whereas avoidance of parameters along the y-axis are represented by negative values along the x-axis. Study was conducted July 15–September 30, 2014, in the Whittell Forest and Wildlife Area, NV, USA

While all seasons included multiple models with Δ AICc scores< 2, some of these top models included uninformative parameters [51, 52]. During both the bitterbrush seed and interval seasons, there were multiple models with Δ AICc scores< 2, but the additional parameters in lower ranked models were uninformative and did not improve model fit. The pine season had four models with a delta AIC < 2, with the fourth ranked model containing uninformative parameters. Since the remaining top three models all contained informative parameters and parameters in the top ranked model were not a subset of lower ranked models, the top three models were averaged. In some models across seasons, distance to the burrow or refuge was best modeled as a quadratic relationship which indicated selection of an intermediate distance from the burrow or points of refuge. The top model for the bitterbrush season included a quadratic relationship for distance to burrow (linear term: β = − 1.5257 SE = 0.0891, squared term: β = 0.7973 SE = 0.0558), selection for bitterbrush canopy cover (β = 0.5929 SE = 0.1510), and selection for open canopy cover (β = 0.7487 SE = 0.0537) (Fig. 1). Additionally, individuals used locations closer to refuge areas than predicted by availability (β = − 0.7318 SE = 0.0558). During the season in which no seeds were available, the top model included a quadratic relationship for distance to refuge areas (linear term: β = 0.058 SE = 0.1638, squared term: β = − 0.1623 SE = 0.0686), avoidance of bitterbrush canopy cover (β = − 0.2373 SE = 0.0647), and avoidance of open canopy cover (β = − 0.1494 SE = 0.0439). Additionally, individuals used locations closer to the burrow than predicted by availability (β = − 0.8450 SE = 0.0298). The three models averaged for pine season all contained quadratic relationships for distance to burrow (linear term: β = − 4.3109 SE = 0.1928, squared term: β = 3.2553 SE = 0.1693). The top model additionally included a quadratic relationship for distance to refuge areas (linear term: β = − 0.2534 SE = 0.1719, squared term: β = 0.2951 SE = 0.1890).

**Fig. 2:**
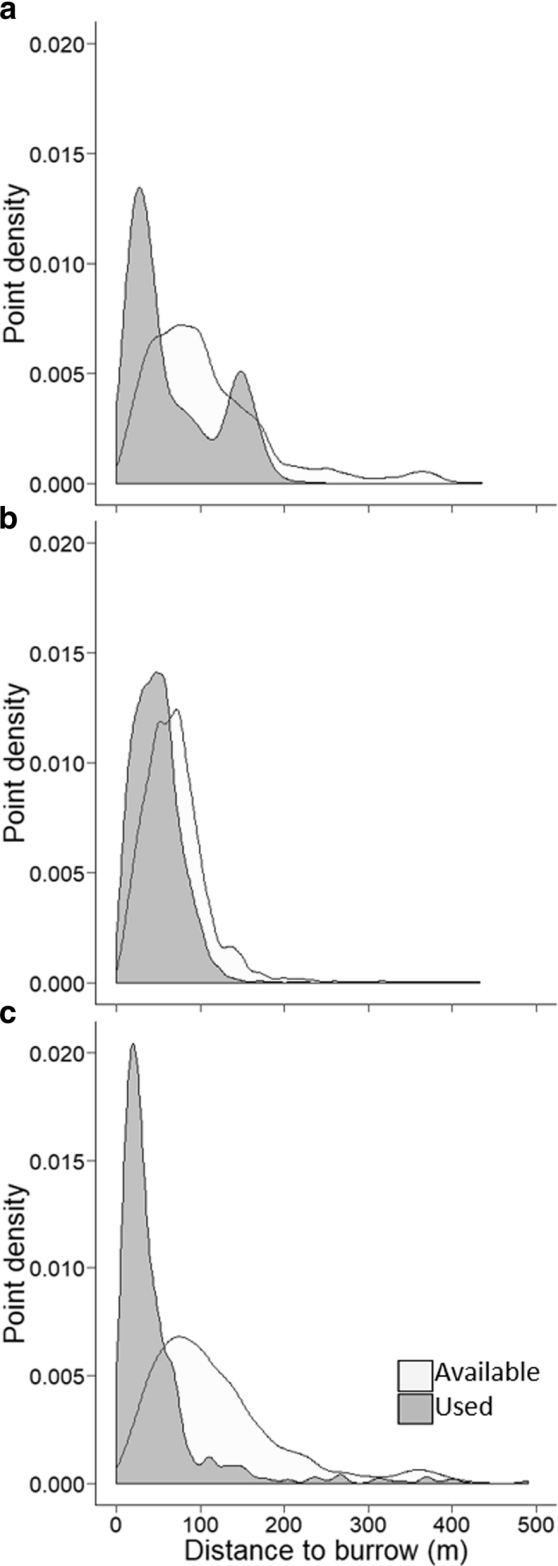
Density plots of GPS logged points from *Callospermophilus lateralis* depicting distance traveled from the burrow (m) during three seasons of food availability: **a**) Bitterbrush, **b**) Interval (no seed available), and **c**) Pine. The dark gray indicates used points (gained from GPS loggers) and the light gray indicates randomly generated available points. Study was conducted July 15–September 30, 2014, in the Whittell Forest and Wildlife Area, NV, USA

**Fig. 3:**
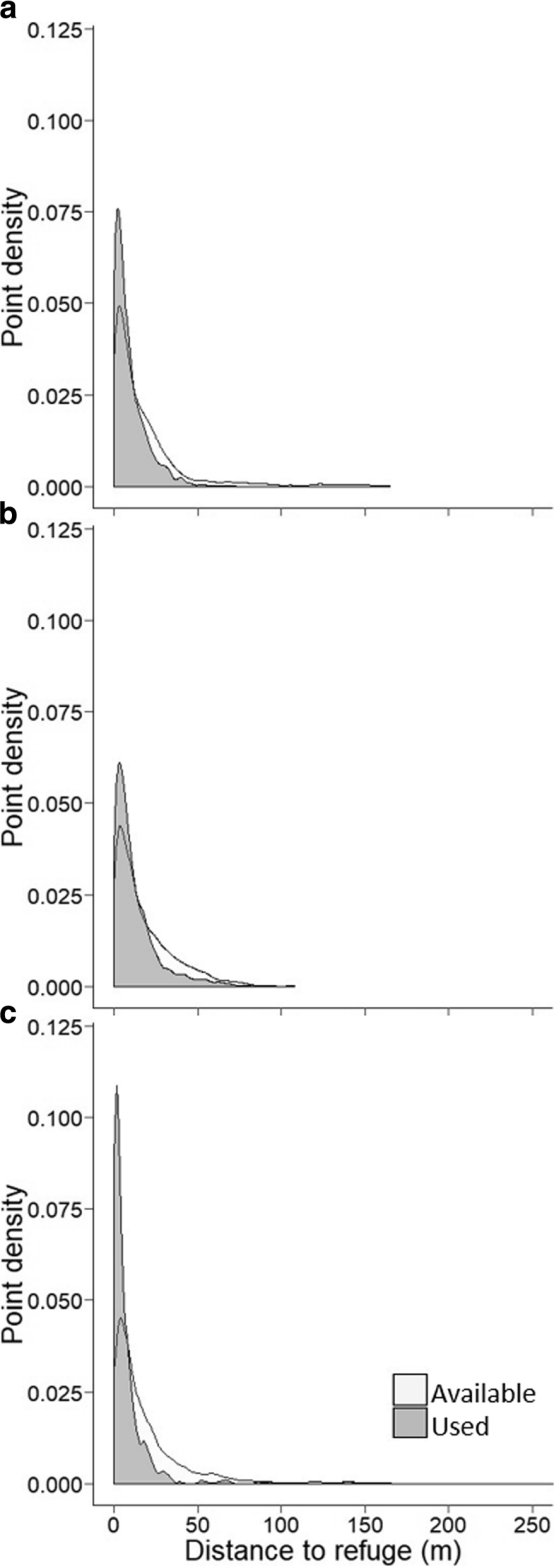
Density plots of GPS logged points from *Callospermophilus lateralis* depicting distance traveled from the refuge (m) during two seasons of food availability: **a**) Bitterbrush and **b**) Interval (no seed available), and **c**) Pine. The dark gray indicates used points (gained from GPS loggers) and the light gray indicates randomly generated available points. Study was conducted July 15–September 30, 2014, in the Whittell Forest and Wildlife Area, NV, USA

Distance to the burrow was best modeled as a quadratic relationship for both seasons that seeds were available. For both seasons, this quadratic relationship indicated higher use of locations closest to the burrow compared to available locations (Fig. 2). Additionally, during bitterbrush season this quadratic function indicated increasing use of locations at intermediate distances from the burrow compared to available locations. For pine season, this function indicated a slight increase in use of locations farthest from the burrow, although overall individuals remained closer to the burrow. Quadratic relationships for distance to refuge areas were also observed for the season of no seed availability and pine season. When plotted, it was evident that use was highest at locations close to refuge areas compared to available locations (Fig. 3).

During bitterbrush season, individuals selected both open and bitterbrush canopy cover, though they selected open canopy cover slightly more than bitterbrush. During the season of no seed availability, individuals avoided both open and bitterbrush canopy cover. During pine season, individuals showed no significant selection for or avoidance of either open or bitterbrush canopy cover relative to pine canopy cover.

## Discussion

The ability to balance conflicting demands is key to surviving in highly competitive and risky landscapes [13, 20, 57]. For *C. lateralis*, this balance means optimizing foraging efforts to meet energetic requirements while also minimizing the risk of predation and loss of food stores to pilferers. Individuals modified travel distances from the burrow and points of refuge, as well as differentially selected vegetative canopy cover by season that could be indicative of individuals attempting to balance these conflicting demands. Contrary to our first prediction, during seasons of seed availability there was evidence of use of locations at intermediate and farther distances from the burrow, in contrast to the season of no seed availability when individuals selected distances closer to the burrow. As predicted, selection for proximity to refuge occurred during seasons of seed availability when individuals foraged away from the burrow. Finally, selection for vegetative canopy cover reflected seed availability by season.

Risk of starvation and predation are two of the greatest threats to the survival of *C. lateralis* as well as other small rodents [58]. For central-place foraging species, the burrow acts as a protective shelter from predators [59–61], but for *C. lateralis*, proximity to the burrow also is important to protect the larder from pilferers (personal observation, KLH). When and how individuals choose to maintain foraging activity in risky environments (i.e. at great distances from the burrow) is dependent on seed availability and individual energy requirements [62–64]. In circumstances when energy requirements are high, the need to forage may even supersede predator avoidance [65, 66]. While a quadratic relationship best represented distance traveled from the burrow for both seasons of seed availability, there was variation in the proximity of those distances traveled from the burrow between seasons (see Fig. 2). In particular, individuals displayed strong selection of intermediate locations (100–200 m) away from the burrow in comparison to pine season. Bitterbrush is the first seed to become available in the summer and individuals are obligated to travel from the burrow to bitterbrush patches where seeds are available.

In contrast, during pine season individuals generally remained closest to the burrow (see Fig. 2). During years with high pine mast, pine seeds predominate other seed species in *T. amoenus* winter larders in Little Valley [25], indicating these seeds are highly valuable for winter survival. Unlike bitterbrush seeds, pine seeds are dispersed randomly by wind events and individuals are not obligated to travel long distances if they encounter seeds closer to the burrow. Pilfering pressure may also be higher on individuals at this time during the year, as the larder becomes larger and more valuable [25, 34]. In a study testing the pilfering rates of red squirrels (*Tamiasciurus hudsonicus*), squirrels with larger middens were pilfered from at a higher rate than squirrels with smaller middens [67]. Individuals may return to their burrows for shelter during bitterbrush season rather than to defend the larder, then switch to a larder-defense strategy during pine season when the larder is more valuable. Although some exceptionally long trips from the burrow were logged during pine season, these trips may likely be explained by the increased value of pine seeds late in the year. When seeds nearest to the burrow become depleted, it becomes necessary to make long trips to accrue enough food to survive winter torpor.

Small mammal species must adopt resource use strategies that balance foraging and predator avoidance [22, 68]. This balance may best be achieved for *C. lateralis* by concentrating activity at locations nearer to the burrow when possible or at intermediate distances from the burrow when seed sources are patchily distributed as in bitterbrush season. Following bitterbrush season, individuals may simply be less inclined to travel frequently from the burrow when encounter rate with seeds has drastically decreased. Consistent with optimal foraging theory, a forager’s harvest rate should decrease as resources in a patch become depleted [69].

If individuals are obligated to travel farther from the burrow to gather seeds, proximity to refuge locations becomes important to reduce predation risk. Although there are certainly more refuge areas than were detected in the study, overall, proximity to locations of refuge appeared to allow individuals to maximize their time foraging while simultaneously reducing their risk of predation. Many other central-place foraging species have been shown to forage near areas of cover when away from the nest or burrow [22, 68]. As explained by previous studies using giving-up densities, animals are less likely to abandon foraging entirely in risky landscapes if they have ready access to refuge [70–73]. This allows individuals to meet their energetic requirements while dually minimizing predation risk.

Proximity to refuge also may enable individuals to use riskier corridors. Although it may seem surprising that individuals select open canopy cover during the bitterbrush season, there are several reasons why individuals may want to use open canopy cover types while foraging. In particular, antelope bitterbrush grows most commonly in shrub stands with little to no overstory cover [74]. Additionally, traveling through open canopy is also more cost effective and may allow them to return to bitterbrush patches and harvest seeds more quickly [75]. Larger-bodied members of the granivore guild have even been shown to exploit riskier open areas to avoid competitive exclusion and are able to do so because of their increased locomotive ability [76]. Unlike their smaller-bodied competitors, larger desert rodents have been shown to select open canopy foraging patches of higher predation risk if the quality of seed is high [77]. This differential selection of foraging patches also promotes coexistence between species within the granivore guild [78].

When seeds were not available, individuals no longer selected habitat patches beneath open canopy (see Fig. 1). When individuals are less active in foraging away from the burrow, they should conserve energy, protect their larder, and avoid predation. During pine season, bitterbrush and open canopy were neither selected nor avoided but used in proportion to their availability. Pine seeds are scattered at random by the wind, so seed encounter rate could be just as likely near to the burrow as well as farther from the burrow until availability of seeds near the burrow are depleted.

The conclusions made in this study are limited by small sample sizes, but to our knowledge this is the first study to use GPS data loggers on species with such a small body size. Nevertheless, this study has shown that movement data can still have great explanatory power when uncovering how individuals make decisions to reduce risk and how these decisions changed as related to changes within their habitat. This is particularly relevant to species for which basic ecological data is already known, such as *C. lateralis*. Additionally, while there are great benefits to using GPS technology, it is many times more expensive than traditional VHF telemetry. Additionally, as occurred in this study, burrowing animals present unique challenges to both GPS and telemetry equipment. Antennas can snap off in tunnels and animals can lose the logger altogether while squeezing through narrow places. Loggers were shed inside burrow tunnels on three occasions and were retrieved if they still emitted a VHF signal. If the telemetry antenna was snapped off, the logger could not be tracked and retrieved. Two study animals disappeared from the site, mostly likely due to predation events, though a logger from one individual was found on top of a boulder inside the study site. Overall, from field observation, loggers did not appear to inhibit movement nor behavior of individuals in the field. Normal foraging activity, grooming, and pursuit of competitors was still observed.

## Conclusions

Use of high-resolution location data allowed for a more accurate understanding of movement decisions *C. lateralis* makes in response to fluctuations of food availability and dynamic stressors. When applied to studies of small mammals, these data provide promising unbiased evidence of individual movements decisions as influenced by intrinsic and extrinsic demands. Using GPS technology allows animals the ability to behave normally without the interruption of researchers actively tracking them. GPS also saves time in the field, which can ultimately save money in travel expenditures. As GPS equipment becomes smaller in size, more affordable, and thus more accessible for small mammal studies, conducting such analyses for small mammals should be more feasible.

## Data Availability

The datasets used and/or analyzed during the current study are available from the corresponding author on reasonable request.
